# Epidemiology, patient characteristics, and treatment patterns of patients with narcolepsy in Sweden: a non-interventional study using secondary data

**DOI:** 10.1093/sleepadvances/zpae085

**Published:** 2024-12-24

**Authors:** Anna Giertz, Johan Mesterton, Tanja Jakobsson, Stephen Crawford, Somraj Ghosh, Anne-Marie Landtblom

**Affiliations:** Quantify Research, Stockholm, Sweden; Quantify Research, Stockholm, Sweden; Department of Learning, Informatics, Management and Ethics, Medical Management Centre, Karolinska Institutet, Stockholm, Sweden; Takeda Pharma AB, Stockholm, Sweden; Takeda Development Center Americas, Inc., Cambridge, MA, USA; Takeda Development Center Americas, Inc., Cambridge, MA, USA; Department of Medical Sciences, Uppsala University, Uppsala, Sweden; Department of Biochemical and Clinical Sciences, Linköping University, Linköping, Sweden

**Keywords:** incidence, narcolepsy, prevalence, Sweden, treatment patterns

## Abstract

**Study Objectives:**

To estimate the prevalence and incidence and evaluate the treatment patterns of patients diagnosed with narcolepsy in specialist care in Sweden.

**Methods:**

This non-interventional retrospective longitudinal study used Swedish register data from 2010 to 2020 and included patients diagnosed with narcolepsy (either type 1 or type 2), recorded in specialist outpatient and inpatient care from January 2015 to December 2019. All patients received an index date corresponding to the date of the first narcolepsy diagnosis.

**Results:**

The prevalence and incidence of narcolepsy were 14.7/100 000 and 0.9/100 000 individuals, respectively, with a greater proportion of females than males. The study included 1846 prevalent narcolepsy patients of either type, of which 466 were incident. The majority of prevalent patients (87.9%) were prescribed narcolepsy-related treatment at index with stimulants being the most common treatment. Both in the years before and after index, the most used medication by prevalent patients was stimulants (42.4% and 54.8%, respectively). Among incident patients, stimulants were the most common drug in the year after index (57.0%). Treatment switching following index was frequent and a large share of incident patients who started on modafinil were switched to stimulants.

**Conclusions:**

The prevalence of narcolepsy was lower than previously reported and was higher in females than in males; incidence was comparable throughout the study period. At index, not all patients used narcolepsy-related medications, potentially indicating a hesitance towards treatment and/or a need for faster initiation of treatment following index. Many patients were switched from the treatment they first initiated after diagnosis, which might be due to a lack of efficacy and/or unacceptable side effects.

## Graphical Abstract

**Graphical Abstract:**
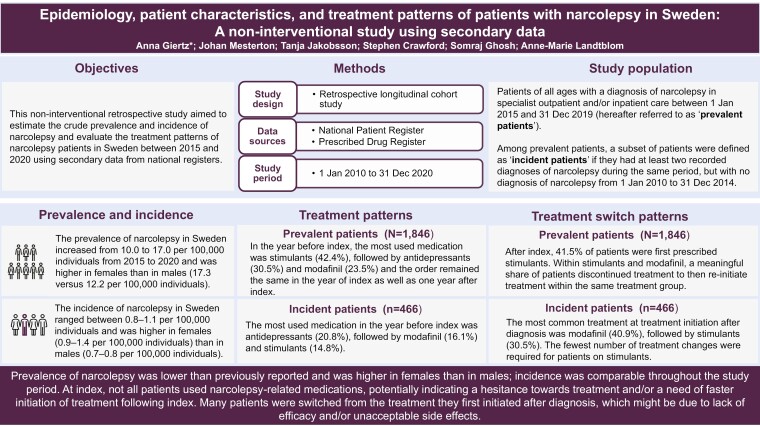

**Statement of Significance**

This was a non-interventional retrospective cohort study which estimated the prevalence and incidence of narcolepsy and evaluated the treatment patterns of patients with narcolepsy in Sweden using Swedish register data from specialist outpatient and inpatient care from 2015–2019. These registers have full coverage of the Swedish population, providing unmatched quality and completeness for decision-making. Based on specialist care data, prevalence was lower than previously reported and incidence was comparable throughout the study period. At the time of the first narcolepsy diagnosis, not all patients used narcolepsy-related medications, potentially indicating a need for faster initiation of treatment following the index. Many patients were switched from the treatment they first initiated after diagnosis, which might be due to the lack of efficacy of available treatments.

Narcolepsy is a rare, chronic, central nervous system disorder of hypersomnolence characterized by excessive daytime sleepiness, which is associated with cataplexy, hypnagogic or hypnopompic hallucinations, sleep paralysis, and disrupted nighttime sleep [1, 2]. There are two major types of narcolepsy: narcolepsy type 1 (NT1) and narcolepsy type 2 (NT2), the former distinguished by symptoms of cataplexy and a loss of orexin-producing neurons in the lateral hypothalamus [2, 3]. A typical cataplectic attack involves a sudden and spontaneous loss of voluntary muscle tone during wakefulness that is evoked by emotional situations (either negative or positive) [4].

Reported global prevalence estimates of narcolepsy range from 0.2 to 65.4 per 100 000 individuals, barring one study which reported an estimate of 79.4 per 100 000 individuals [5, 6]. The estimated overall prevalence of narcolepsy in Europe has been reported to be 40.6 per 100 000 individuals (NT1: 19.7; 95% confidence interval [CI]: 5.1 to 34.3 and NT2: 20.9; 95% CI: 5.9 to 36.0) based on a multi-country study utilizing a random sample of participants which was representative of the European population [7]. The prevalence of narcolepsy in the US population increased by 14% between 2013 (38.9 per 100 000 individuals) and 2016 (44.3 per 100 000 individuals) based on a retrospective cohort study which used Symphony Health data (2013–2016) [8]. However, Scheer et al. reported a substantially higher prevalence of narcolepsy (79.4 per 100 000 individuals) in the United States based on data from a 2008 to 2010 US healthcare claims database [5]. This was an outlier caused by the case ascertainment algorithm used in the study producing higher sensitivity and lower specificity than other reported studies. Such a variation in prevalence estimates might be attributed to differences in methodology, study periods, diagnostic criteria, and study designs [6, 9].

The overall prevalence of narcolepsy in Sweden is not known. However, in a recent study focusing on the treatment of narcolepsy in Sweden between 2005 and 2017, 2508 patients were identified using the Swedish National Patient Register (NPR), representing a prevalence of around 25 per 100 000 individuals [10]. It is also known that after the H1N1 pandemic, including mass vaccination with Pandemrix (2009–2010), there was an increase in the incidence of narcolepsy [11, 12].

Narcolepsy cannot be cured and even when treated, it can be psychosocially devastating. Affected individuals often experience tiredness and apathy, which may result in social isolation [13]. A range of medications are available for the treatment of narcolepsy. Currently, these include drugs that promote wakefulness, i.e. for excessive daytime sleepiness (modafinil, methylphenidate, amphetamines, and others), and adrenergic and serotonergic reuptake inhibitors for cataplexy. Pitolisant and sodium oxybate also promote wakefulness and prevent cataplexy [1, 14–16].

Currently, only a few observational studies on narcolepsy describe the clinical characteristics, epidemiology, treatment patterns, societal outcomes, and overall survival in a real-world setting in Sweden [10, 17]. This non-interventional retrospective study aimed to estimate the crude prevalence and incidence of narcolepsy and evaluate the treatment patterns of narcolepsy patients in Sweden between 2015 and 2020 using secondary data from national registers.

The Swedish administrative databases used in this study have full coverage of the Swedish population (approximately 10 million individuals). It is compulsory to report to these registers, and patients remain included in them from birth until either their death or emigration, which minimizes loss to follow up and makes cohorts representative of the general population, providing unmatched quality and completeness for decision-making. Individual identifiers allow for linkage across registers for longitudinal studies of large cohorts, making it feasible to analyze data on healthcare visits, diagnoses and procedures, prescribed treatments, long-term sick leave, demographics, and causes of death.

## Methods

### Study design and patient population

This was a retrospective longitudinal cohort study which used pseudonymized patient-level data from Swedish registers, namely the NPR and the prescribed drug register (PDR; **Table 1**). The study included patients of all ages with at least one primary or secondary diagnosis of narcolepsy (NT1 or NT2, without distinction; ICD-10-SE codes: G47.4, G47.4A, G47.4B, G47.4W, and G47.4X) in specialist outpatient and/or inpatient care between January 01, 2015, and December 31, 2019 (hereafter referred to as “prevalent patients”). Specialist care involves all hospital-associated care but does not include primary care or general practitioner visits. Among prevalent patients, a subset of patients was defined as “incident patients” if they had at least two recorded diagnoses of narcolepsy (of which one was primary) during the same period, but with no diagnosis of narcolepsy from January 01, 2010, to December 31, 2014. These two cohorts were based on different aims: capturing as many narcolepsy patients as possible, which formed the prevalent cohort, and ensuring the patient cohort consisted of confirmed narcolepsy cases with stricter inclusion criteria (requiring a minimum of two diagnoses) which formed the incident cohort.

**Table 1.** Summary of Key National Registers Used in This Study

Type of data	Source registers	Details
Inpatient care	National Patient Register (NPR)	Includes the dates and diagnoses associated with all in-patient specialist care in Sweden, as well as demographics such as patient’s age and sex. Data are available from 1964 and onward.
Outpatient care	National patient register (NPR)	Includes the dates and diagnoses associated of all out-patient specialist care in Sweden (does not include primary care), as well as demographics such as patient’s age and sex. Data is available from 1997 and onward.
Prescriptions	Prescribed drug register (PDR)	All prescriptions dispensed by patients at Swedish pharmacies are included in the registry. The data includes date of prescription and dispensation, formulation, amount dispensed, defined daily dose, and others from July 2005 and onward.

**Figure 1.**
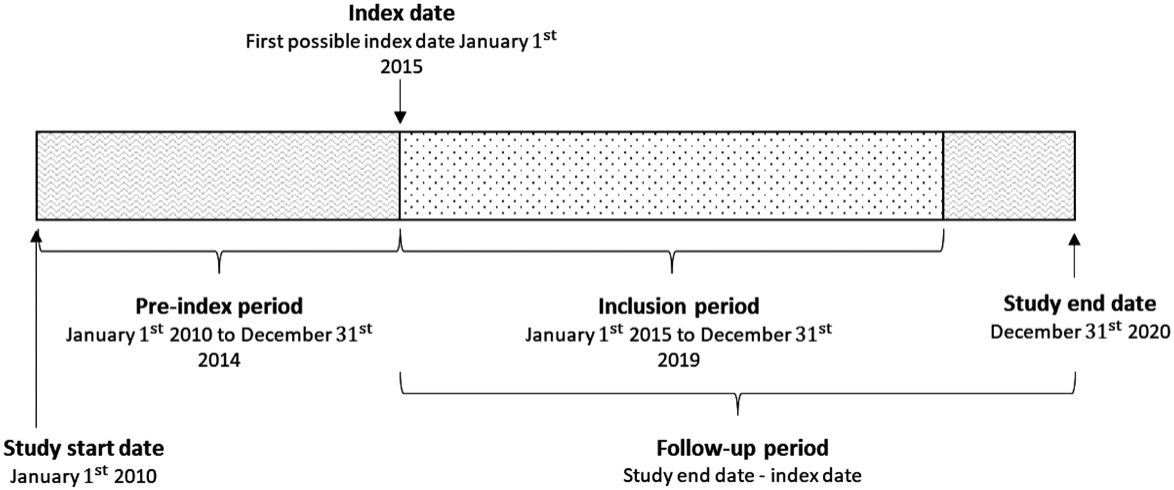
Schematic overview of the study period.

The study period ranged from January 01, 2010, to December 31, 2020 (**Figure 1**). All patients received an index date corresponding to the date of first narcolepsy diagnosis in the inclusion period (prevalent patients who were diagnosed with narcolepsy before the start of the inclusion period had a common index date, January 01, 2015). The look-back period consisted of the 3 years prior to the index date, which was used to identify comorbidities and construct the comorbidity index. Patients were censored from the study in the event of death or end of the study period. All patients had follow-ups from the index date until death or the end of the study period.

### Study objectives and definitions

#### Prevalence and incidence of narcolepsy in Sweden.

The point prevalence and incidence of narcolepsy in Sweden were crudely estimated using data from specialist outpatient and inpatient care on December 31 of each year from year 2015 to 2020. Prevalence was defined as the ratio between the number of unique individuals in the study cohort in a specific year divided by the population on December 31 of the same year, multiplied by 100 000. Incidence was defined as the number of new cases (no prior diagnosis) each year, divided by the population at the start of the same year, multiplied by 100 000.

#### Demographics and characteristics of patients with narcolepsy.

The age and sex of patients were investigated along with comedications at index. Comedications were measured as pharmacy-dispensed medications (See Supplementary Table 1 for ATC-codes of comedications) in the 3 years prior to index date.

#### Treatment patterns.

 The medications dispensed to the prevalent and incident patients were assessed 1 year before and after index as well as in the year of index. All the medications were considered/assessed separately although in clinical practice, patients are likely to receive a combination of treatments.

#### Treatment switch patterns.

Treatment switch patterns were assessed at any time following the index date. the switch was defined as the first dispensation of a narcolepsy-related treatment that differed from the previous narcolepsy-related treatment. The included treatments were based on recent Swedish treatment recommendations (modafinil, sodium oxybate, melatonin, antidepressants, stimulants, and pitolisant) [18]. For details on medications included in the respective medication group (**Supplementary Table 1**).

### Data analysis

The data management and the descriptive analyses were performed using R version 4.0 and Stata version 16. Results were provided for the entire narcolepsy population, which included patients with both NT1 and NT2 without distinction between the two. For demographic profile, treatment pattern and treatment switch-related data in patients, descriptive statistics were used. The results were presented as frequencies and percentages for categorical outcome variables, while continuous variables were described with means and standard deviations (SD). The flows of patients among different treatment options (treatment switch) were presented as Sankey diagrams.

## Results

### Prevalence and incidence of narcolepsy in Sweden

**Figure 2.**
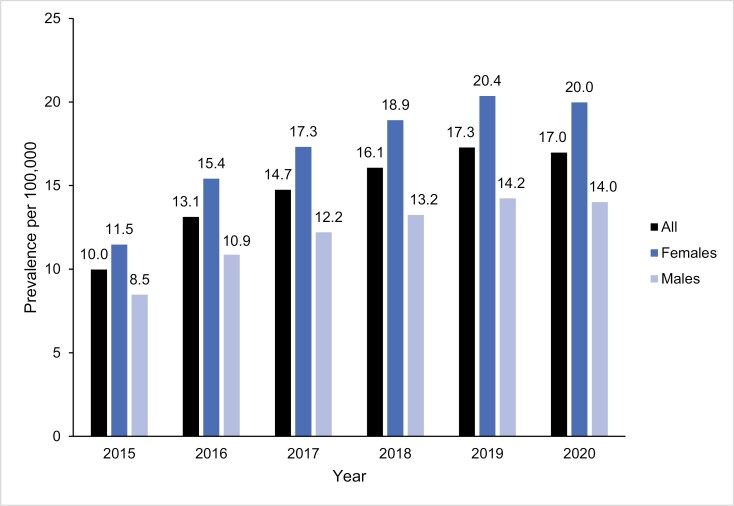
Prevalence of narcolepsy in specialist care in Sweden between 2015 and 2020.

Based on data from specialist outpatient and inpatient care, the prevalence of narcolepsy in Sweden increased from 10.0 to 17.0 per 100 000 individuals from 2015 to 2020 with an average of 14.7 per 100 000 individuals during the study period (**Supplementary Table 2**). The prevalence was higher in females than in males (17.3 vs. 12.2 per 100 000 individuals) and the difference between prevalence in males and females increased over the course of the study, from 3.0 to 6.0 per 100 000 from 2015 to 2020, (i.e. female/male ratio of 1.35 to 1.43; **Figure 2**).

**Figure 3.**
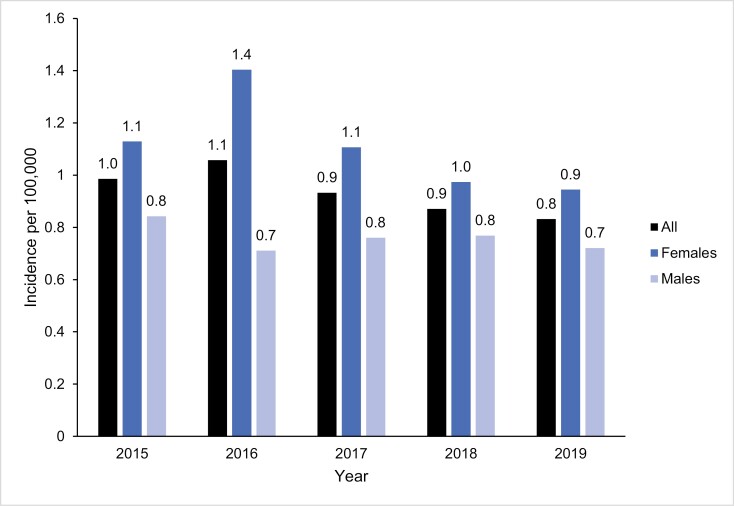
Incidence of narcolepsy in specialist care in Sweden between 2015 and 2020.

The incidence of narcolepsy in Sweden ranged between 0.8 and 1.1 per 100 000 individuals during the study period with an average of 0.9 per 100 000 individuals (**Figure 3**). Incidence was comparable over all the years but slightly decreased over the years of the study (1.0 to 0.8 per 100 000 individuals from 2015 to 2019). The incidence was higher in females (0.9–1.4 per 100 000 individuals) than in males (0.7–0.8 per 100 000 individuals) for all years of the study period (**Supplementary Table 3**).

### Demographics and characteristics of patients with narcolepsy

A total of 1846 prevalent patients were identified from specialist outpatient and inpatient care, of which 466 were classified as incident. Mean age of prevalent patients at index was 38.1 (SD: 22.2) years and of incident patients 31.0 (SD: 18.4) years. The sex distribution was similar between the prevalent (58.3% females) and incident (59.2% females) patients (**Table 2**). The number of prevalent and incident patients on any narcolepsy-related treatment at index was 87.9% and 91.2%, respectively, while 12.1% and 8.8% of patients did not receive any narcolepsy-related treatment (**Table 2**). The most common narcolepsy-related medication dispensed to prevalent and incident patients at index were stimulants (prevalent patients: 55.3%; incident patients: 54.5%) followed by modafinil (prevalent patients: 34.0%; incident patients: 53.4%).

**Table 2.** Demographic Profile and Characteristics of Narcolepsy Patients Identified in Specialist Care Data in Sweden between Years 2015 and 2020

	Number of patients (%)	
Patient demographics at index*	Prevalent patients (*N* = 1846)	Incident patients** (*n* = 466)
Male	769 (41.7)	190 (40.8)
Female	1077 (58.3)	276 (59.2)
Age at index, years (mean [SD])	38.1 (22.2)	31.0 (18.4)
*Age distribution at index*		
≤18 years	369 (20.0)	132 (28.3)
19–29 years	522 (28.3)	161 (34.5)
30–39 years	225 (12.2)	50 (10.7)
40–49 years	214 (11.6)	52 (11.2)
50–59 years	116 (6.3)	18 (3.9)
60–69 years	134 (7.3)	24 (5.2)
70–79 years	168 (9.1)	20 (4.3)
≥80 years	98 (5.3)	9 (1.9)
Comedications at index*		
Total number of patients with any narcolepsy-related dispensation	1623 (87.9)	425 (91.2)
Stimulants	1020 (55.3)	254 (54.5)
Modafinil	628 (34.0)	249 (53.4)
Pitolisant	≤5 (-)	≤5 (–)
Antidepressants	681 (36.9)	185 (39.7)
Sodium oxybate	223 (12.1)	45 (9.7)
Benzodiazepine derivatives	210 (11.4)	40 (8.6)
Melatonin receptor agonists	138 (7.5)	51 (10.9)
Other hypnotics and sedatives	74 (4.0)	13 (2.8)
Zolpidem/ CR	70 (3.8)	10 (2.2)
Baclofen	6 (0.3)	≤5 (–)

*The index date was a proxy for diagnosis; for incident patients, the index date was the first diagnosis of narcolepsy within the study period with a prior diagnosis-free period of five years; for prevalent patients, the index date was the first diagnosis of narcolepsy within the study period.

**Note that the incident patients were a subset of prevalent patients.

### Treatment patterns

#### Prevalent patients.

In the year before index, the most used medication was stimulants (42.4%), followed by antidepressants (30.5%) and modafinil (23.5%) and the order remained the same in the year of an index as well as 1 year after the index. From the year before index to the year of index, the use of stimulants increased from 42.4% to 55.3% and antidepressants from 30.5% to 36.9% (**Table 3**).

**Table 3.** Medications Used in the Year Before, of and After Index in the Prevalent Narcolepsy Patients (*N* = 1846) Identified in Specialist Care Data in Sweden Between Year 2015 and 2020

	Year 1 before index			Year of index			Year 1 after index		
Medications	*N* ^1^	%	Mean ^2^	*N* ^1^	%	Mean ^2^	*N* ^1^	%	Mean ^2^
Modafinil	433	23.5	1.2	628	34.0	1.7	459	25.3	1.3
Stimulants	783	42.4	3.1	1020	55.3	4.4	994	54.8	4.2
Antidepressants	563	30.5	1.8	681	36.9	2.0	635	35.0	2.0
Sodium oxybate	121	6.6	0.4	223	12.1	0.7	241	13.3	0.8
Pitolisant	0	0.0	0.0	≤5	—	0.0	8	0.4	0.0
Benzodiazepine derivatives	177	9.6	0.8	210	11.4	0.8	192	10.6	0.9
Melatonin receptor agonists	92	5.0	0.1	138	7.5	0.2	138	7.6	0.2
Other hypnotics and sedatives	60	3.3	0.1	74	4.0	0.1	70	3.9	0.1
Zolpidem/ CR	63	3.4	0.1	70	3.8	0.2	62	3.4	0.2
Baclofen	≤5	—	0.0	6	0.3	0.0	17	0.9	0.0

^1^Number of patients with at least one dispensation of medication;

^2^Number of dispensed medications per patient and year.

#### Incident patients.

The most used medication in the year before the index was antidepressants (20.8%), followed by modafinil (16.1%) and stimulants (14.8%). The use of antidepressants, modafinil, and stimulants increased in the year index to 54.5%, 53.4%, and 39.7%, respectively (**Table 4**).

**Table 4.** Medications Used in the Year Before, of and After Index in the Incident Narcolepsy Patients (*n* = 466) Identified in Specialist Care Data in Sweden Between Years 2015 and 2020

	Year 1 before index			Year of index			Year 1 after index		
Medications	*N* ^1^	%	Mean ^2^	*N* ^1^	%	Mean ^2^	*N* ^1^	%	Mean ^2^
Modafinil	75	16.1	0.7	249	53.4	2.4	149	32.2	1.5
Stimulants	69	14.8	0.9	254	54.5	4.7	264	57.0	4.4
Antidepressants	97	20.8	1.0	185	39.7	1.8	162	35.0	1.7
Sodium oxybate	≤5	—	0.0	45	9.7	0.6	47	10.2	0.6
Pitolisant	≤5	—	0.0	≤5	—	0.0	≤5	—	0.0
Benzodiazepine derivatives	29	6.2	0.4	40	8.6	0.6	32	6.9	0.6
Melatonin receptor agonists	17	3.6	0.1	51	10.9	0.4	47	10.2	0.3
Other hypnotics and sedatives	12	2.6	0.1	13	2.8	0.1	19	4.1	0.1
Zolpidem/ CR	≤5	—	0.0	10	2.1	0.1	9	1.9	0.1
Baclofen	≤5	—	0.0	≤5	—	0.0	≤5	—	0.1

^1^Number of patients with at least one dispensation of medication;

^2^Number of dispensed medications per patient and year.

For details see Supplementary Table 1.

### Treatment switch patterns

#### Prevalent patients.

**Figure 4.**
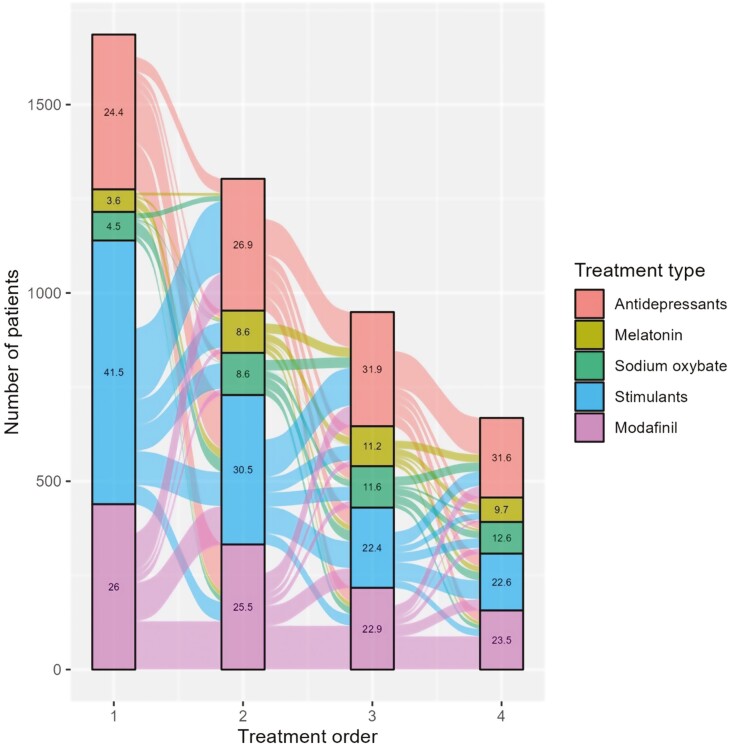
Treatment switch pattern in prevalent patients.

After the index, 41.5% of patients were first prescribed stimulants. This was also the medication group with the fewest treatment changes. Within stimulants and modafinil, a meaningful share of patients discontinued treatment to then reinitiate treatment within the same treatment group (**Figure 4**).

#### Incident patients.

**Figure 5.**
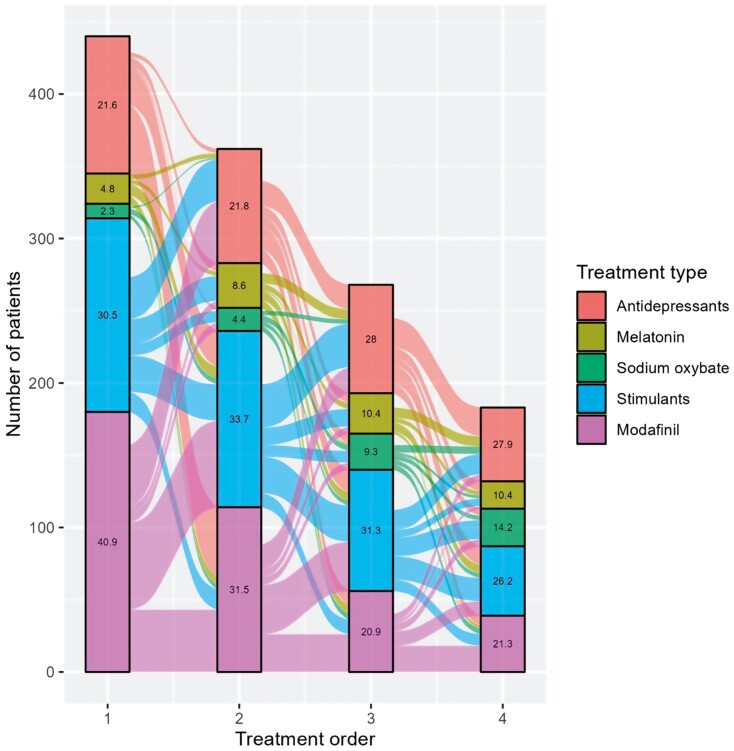
Treatment switch pattern in incident patients.

The most common treatment at treatment initiation after diagnosis was modafinil (40.9%), followed by stimulants (30.5%). The fewest treatment changes were required for patients on stimulants. However, there was an increase in the share of patients switching to antidepressants with each subsequent treatment switch, indicating that patients transitioned/switched from not using antidepressants to using them as their treatment regimen. A large share of patients who started on modafinil were switched to stimulants. Within stimulants and modafinil, a large share of patients discontinued to then re-initiate treatment within the same treatment group (**Figure 5**).

## Discussion

The present study aimed to describe narcolepsy patients identified in specialist outpatient and inpatient care in Sweden from year 2015 to 2020. The prevalence of narcolepsy patients was 14.7 per 100 000 individuals (corresponding to 1470 patients) which was lower than that reported in a recent Swedish study by Gauffin et al., 2022 (25 per 100 000 individuals) which used the same data sources and identification strategy. The difference in prevalence could be due to the different time periods studied (2015–2020 compared to 2005–2017 in Gauffin et al. 2022).

The prevalence presented in this study might be too low as it is possible that patients who were adequately diagnosed and treated before the identification period did not seek care regularly and were therefore not captured in data during the identification period. Moreover, the NPR captures information on specialized care but does not include primary care data [19]. It was also observed that the prevalence increased over the study period, and the main share of prevalent patients were identified and included in the study population in 2015. This is in large part due to the study design, as all prevalent patients with a diagnosis prior to the inclusion period received a common index date of January 01, 2015.

The estimated prevalence of narcolepsy was three to five times lower compared to US studies [5, 8]. However, there is a large variation in epidemiology estimates among US studies and the findings of this study cannot be directly compared with those studies because of differences in data sources and methodology. According to a meta-analysis assessing the incidence and prevalence of narcolepsy worldwide, the prevalence of narcolepsy was found to be higher in studies conducted in the United States (odds ratio [OR]: 11.5; *p* < .001) and Asia (OR: 17.5; *p* < .001) than in Europe [6].

In this study, the prevalence of narcolepsy was higher in females than in males, which is in concordance with the Sweden-based studies by Gauffin et al. (56.7% vs. 43.3%) [10] and Wandell et al. (female–male ratio for Swedish‐born patients: 1.82, and for foreign‐born patients: 1.02) [17] as well as US studies by Acquavella et al. (60% vs. 40%) [8] and Scheer et al. (50% greater prevalence than males) [5]. This may be explained by females using the health care system more and seeking care for narcolepsy more often than males [5, 10]. In contrast, some studies reported equal prevalence in males and females but observed that females were more likely than males to experience delays in diagnosis or remain undiagnosed at any given time [20, 21].

The incidence of narcolepsy was 0.9 per 100 000 individuals in the present study, which was in line with a German study by Kallweit et al., 2022 (12-month incidence: 0.8 per 100 000 individuals) [22]. The number of patients identified decreased over the study period and the only exception to the trend in identification was in 2016. This might be due to the fact that the study was performed several years after the H1N1 pandemic or to changes in clinical practice regarding new more strict criteria for diagnosing narcolepsy. These new criteria include the option to measure cerebrospinal fluid (CSF) orexin levels through lumbar puncture [23].

The incidence of narcolepsy varies across geographical locations and time periods [6]. An increased incidence of narcolepsy after the H1N1 pandemic 2009–2010 has been reported in Northern Europe including Sweden, Norway and Finland [24–27], and China [28–30]; however, South Korea [31], and the United States [32] were not impacted. The Swedish Medical Products Agency conducted an early case inventory study to assess the association between the Pandemrix vaccine and the development of narcolepsy with cataplexy. They found that the incidence rate was higher in vaccinated individuals than in unvaccinated individuals (4.2 versus 0.6 per 100 000 person-years) and had a relative risk of 6.6 and an absolute risk of 3.6 additional cases per 100 000 vaccinated cases [33]. Interestingly, a recent data analysis indicates that also the virus H1N1 per se may have increased the prevalence in Sweden [12]

A majority of prevalent patients were treated with narcolepsy-related medications at index (87.9%) and the same was true for the subset of patients that were incident (91.2%). Overall, 223 (12.1%) patients did not receive any narcolepsy-related treatment at index. One potential explanation for this could be that these patients received treatment that was not defined as narcolepsy-related in the present study. There is also the possibility that some patients refrained from or discontinued treatment due to the addictive nature of some of the treatment options (e.g. stimulants, sodium oxybate, etc.) or an unfavorable risk–benefit ratio or hesitance to initiate treatment.

Treatment patterns observed in the present study are in line with Gauffin et al. [10]. Stimulants, antidepressants, and modafinil were the most prescribed treatments throughout the study period. A share of incident patients were prescribed narcolepsy-related treatments in the year prior to the index date. This could be due to some medications being used for an indication other than narcolepsy, e.g. antidepressants. However, since the second and third most prescribed medications were modafinil and stimulants, it is also possible that these patients were not incident, but simply had not sought specialist care in the years prior to identification and were therefore misclassified. Treatment switch and prescription of multiple medications for combination treatment is often necessary for long-term management of narcolepsy [34]. This was also evident in this study as treatment switch was frequent among patients. However, patients on stimulants required the fewest treatment changes.

Pitolisant and sodium oxybate, two highly effective medications for the treatment of narcolepsy [15, 35, 36], are not covered by the Swedish reimbursement programme since their cost-effectiveness has not been established. There is still the possibility that, with the healthcare provider’s agreement, the cost of sodium oxybate may be reimbursed at a regional level, but pitolisant is unfortunately still poorly accepted by the healthcare system. It seems plausible that the health provider and/or narcolepsy patients may be unwilling or unable to pay for pitolisant treatment, which could provide an explanation for why usage of this narcolepsy-specific drug is lower than expected.

## Limitations

The epidemiological findings of the present study were based on Swedish data and are therefore not directly generalizable to other countries, as treatment practices and underlying characteristics in the populations may differ. Furthermore, the study reports the diagnosed prevalence rather than the population prevalence, including all patients with a recorded diagnosis of narcolepsy in specialist care but excluding primary care data. However, based on the nature of the disease, patients are expected to be treated in specialist care and so the number of patients missing due to the lack of primary care data is likely limited. A probable cause for underestimation of prevalence in this study is the relatively short period for data collection (from the year 2015 to 2020), so narcolepsy patients who did not receive specialist care during this time period did not appear in the study. This is likely to occur since patient identification was only based on specialist visits, not prescriptions, meaning patients that are adequately treated may not seek care and will therefore not be captured in our analysis. Similarly, patients refraining from treatment will not be captured in our analysis. The main drivers for treatment discontinuation are treatment-related adverse effects, lack of efficacy, pregnancy, or hesitation due to the narcotic nature of the drugs. In older patients, stimulants may in addition interfere with cardiovascular problems. Given that, outcomes were defined using national and population-based health register data, there is an inherent risk of information bias resulting from classification error.

The current study did not investigate prior hypersomnia diagnoses among individuals seeking specialist care for narcolepsy. Additionally, information on narcolepsy sub-types (NT1/NT2) was available for only a very small subset of the full population, limiting the ability to draw meaningful conclusions due to the small sample size. The ICD-10-SE coding system includes sub-codes that, in theory, could distinguish between NT1 and NT2. However, these sub-codes are relatively new and have not been widely used, making them insufficient for identification. This limitation affects the interpretation of our analyses, as the database only contains sufficient detail to distinguish between NT1 and NT2 for a small (and likely not random) subset of the population.

## Conclusion

The present study provides nationwide data on the epidemiology, characteristics, and treatment patterns of patients diagnosed with narcolepsy in specialist care. In spite of the post-pandemic increase in cases, the prevalence of narcolepsy was lower than previously reported and was higher in females than in males. A meaningful share of patients did not use any narcolepsy-related treatment at index, potentially indicating a need for faster initiation of treatment following disease onset or hesitance of patients to initiate treatment due to an unfavorable benefit-risk profile. Many patients were switched from the treatment they first initiated after diagnosis, which suggests that the currently available treatment options for narcolepsy are not sufficiently effective and/or could exhibit undesirable side effects. Future studies are warranted to understand the reasons for switching pharmaceutical treatments for narcolepsy, and to improve the treatment options.

## Supplementary Material

zpae085_suppl_Supplementary_Materials

## Data Availability

No patient-level data can be made available to other researchers due to Swedish data legislation.

## References

[1] Bhattarai J, Sumerall S. Current and future treatment options for narcolepsy: a review. Sleep Sci. 2017;10(1):19–27. doi: https://doi.org/10.5935/1984-0063.20170004 28966734 PMC5611768

[2] Chavda V, Chaurasia B, Umana GE, Tomasi SO, Lu B, Montemurro N. Narcolepsy-a neuropathological obscure sleep disorder: a narrative review of current literature. Brain Sci. 2022;12(11):1473. doi: https://doi.org/10.3390/brainsci12111473 36358399 PMC9688775

[3] AASM. The AASM International Classification of Sleep Disorders – Third Edition, Text Revision (ICSD-3-TR). https://aasm.org/clinical-resources/international-classification-sleep-disorders/. 2023. Accessed June 20, 2023.

[4] Szabo ST, Thorpy MJ, Mayer G, Peever JH, Kilduff TS. Neurobiological and immunogenetic aspects of narcolepsy: implications for pharmacotherapy. Sleep Med Rev. 2019;43:23–36. doi: https://doi.org/10.1016/j.smrv.2018.09.006 30503715 PMC6351197

[5] Scheer D, Schwartz SW, Parr M, Zgibor J, Sanchez-Anguiano A, Rajaram L. Prevalence and incidence of narcolepsy in a US health care claims database, 2008-2010. Sleep. 2019;42(7). doi: https://doi.org/10.1093/sleep/zsz091 31004158

[6] Wang Y, Chen Y, Tong Y, Li C, Li J, Wang X. Heterogeneity in estimates of incidence and prevalence of narcolepsy: a systematic review and meta-regression analysis. Neuroepidemiology. 2022;56(5):319–332. doi: https://doi.org/10.1159/000525282 35820399

[7] Ohayon MM, Dave S, Shah A, Swick T, Cote ML. Prevalence of Narcolepsy Type 1 and Type 2 in representative samples of the general population of North America, Europe and South Korea. Sleep Med. 2022;100:S164. doi: https://doi.org/10.1016/j.sleep.2022.05.444 39983282

[8] Acquavella J, Mehra R, Bron M, Suomi JM, Hess GP. Prevalence of narcolepsy and other sleep disorders and frequency of diagnostic tests from 2013-2016 in insured patients actively seeking care. J Clin Sleep Med. 2020;16(8):1255–1263. doi: https://doi.org/10.5664/jcsm.8482 32807293 PMC7446073

[9] Longstreth W Jr , Koepsell TD, Ton TG, Hendrickson AF, Van Belle G. The epidemiology of narcolepsy. Sleep. 2007;30(1):13–26. doi: https://doi.org/10.1093/sleep/30.1.13 17310860

[10] Gauffin H, Fast T, Komkova A, Berntsson S, Bostrom I, Landtblom AM. Narcolepsy treatment in Sweden: an observational study. Acta Neurol Scand. 2022;145(2):185–192. doi: https://doi.org/10.1111/ane.13532 34611886

[11] Sarkanen TO, Alakuijala APE, Dauvilliers YA, Partinen MM. Incidence of narcolepsy after H1N1 influenza and vaccinations: systematic review and meta-analysis. Sleep Med Rev. 2018;38:177–186. doi: https://doi.org/10.1016/j.smrv.2017.06.006 28847694

[12] Gauffin H, Boström I, Berntsson SG, Kristoffersson A, Fredrikson M, Landtblom A-M. Characterization of the increase in narcolepsy following the 2009 H1N1 pandemic in Sweden. J Clin Med. 2024;13(3):652. doi: https://doi.org/10.3390/jcm13030652 38337347 PMC10856509

[13] NINDS. Narcolepsy. NIH National Institute of Neurological Disorders and Stroke. https://www.ninds.nih.gov/health-information/disorders/narcolepsy. 2023. Accessed June 20, 2023.

[14] Szakacs Z, Dauvilliers Y, Mikhaylov V, et al; HARMONY-CTP study group. Safety and efficacy of pitolisant on cataplexy in patients with narcolepsy: a randomised, double-blind, placebo-controlled trial. Lancet Neurol. 2017;16(3):200–207. doi: https://doi.org/10.1016/S1474-4422(16)30333-7 28129985

[15] Bolin K, Niska PA, Pirhonen L, Wasling P, Landtblom AM. The cost utility of pitolisant as narcolepsy treatment. Acta Neurol Scand. 2020;141(4):301–310. doi: https://doi.org/10.1111/ane.13202 31838740

[16] Boström I, Lindberger O, Partinen M, Landtblom A. Vaccination against swine flu caused narcolepsy in several European countries. Health Risk Anal. 2020;3:182–187.

[17] Wandell P, Fredrikson S, Carlsson AC, Li X, Sundquist J, Sundquist K. Narcolepsy among first- and second-generation immigrants in Sweden: a study of the total population. Acta Neurol Scand. 2022;146(2):160–166. doi: https://doi.org/10.1111/ane.13633 35543223 PMC9544457

[18] Lind J. Sveriges Regioner i Samverkan. Riktlinje för behandling av narkolepsi hos barn och vuxna. Jönköping. 2022.

[19] NPR. National patient register (both inpatient and outpatient): Socialstyrelsen. Patientregistret. https://www.socialstyrelsen.se/statistik-och-data/register/patientregistret/. 2023. Accessed June 20, 2024.

[20] Marsella JL, Sharkey KM. Sex differences in sleep disorders. In: Attarian H, Viola-Saltzman M, eds. *Sleep Disorders in Women*. Current Clinical Neurology. Cham: Humana; 2020:65–81.

[21] Won C, Mahmoudi M, Qin L, Purvis T, Mathur A, Mohsenin V. The impact of gender on timeliness of narcolepsy diagnosis. J Clin Sleep Med. 2014;10(1):89–95. doi: https://doi.org/10.5664/jcsm.3370 24426826 PMC3869076

[22] Kallweit U, Nilius G, Trumper D, Vogelmann T, Schubert T. Prevalence, incidence, and health care utilization of patients with narcolepsy: a population-representative study. J Clin Sleep Med. 2022;18(6):1531–1537. doi: https://doi.org/10.5664/jcsm.9910 35088707 PMC9163623

[23] Riktlinje för behandling av narkolepsi hos barn och vuxna. https://vardpersonal.1177.se/globalassets/nkk/nationell/media/dokument/kunskapsstod/vardriktlinjer/riktlinje-for-behandling-av-narkolepsi-hos-barn-och-vuxna.pdf. 2022. Accessed August 20, 2024.

[24] Heier MS, Gautvik KM, Wannag E, et al Incidence of narcolepsy in Norwegian children and adolescents after vaccination against H1N1 influenza A. Sleep Med. 2013;14(9):867–871. doi: https://doi.org/10.1016/j.sleep.2013.03.020 23773727

[25] Partinen M, Saarenpaa-Heikkila O, Ilveskoski I, et al Increased incidence and clinical picture of childhood narcolepsy following the 2009 H1N1 pandemic vaccination campaign in Finland. PLoS One. 2012;7(3):e33723. doi: https://doi.org/10.1371/journal.pone.0033723 22470463 PMC3314680

[26] Szakacs A, Darin N, Hallbook T. Increased childhood incidence of narcolepsy in western Sweden after H1N1 influenza vaccination. Neurology. 2013;80(14):1315–1321. doi: https://doi.org/10.1212/WNL.0b013e31828ab26f 23486871

[27] Wijnans L, Lecomte C, de Vries C, et al The incidence of narcolepsy in Europe: before, during, and after the influenza A(H1N1)pdm09 pandemic and vaccination campaigns. Vaccine. 2013;31(8):1246–1254. doi: https://doi.org/10.1016/j.vaccine.2012.12.015 23246544

[28] Han F, Lin L, Warby SC, et al Narcolepsy onset is seasonal and increased following the 2009 H1N1 pandemic in China. Ann Neurol. 2011;70(3):410–417. doi: https://doi.org/10.1002/ana.22587 21866560

[29] Wang X, Xiao F, Wang Y, et al Changed epidemiology of narcolepsy before, during, and after the 2009 H1N1 pandemic: a nationwide narcolepsy surveillance network study in mainland China, 1990-2017. Sleep. 2023;46(3).doi: https://doi.org/10.1093/sleep/zsac325 36595587

[30] Wu H, Zhuang J, Stone WS, et al Symptoms and occurrences of narcolepsy: a retrospective study of 162 patients during a 10-year period in eastern China. Sleep Med. 2014;15(6):607–613. doi: https://doi.org/10.1016/j.sleep.2013.12.012 24767723

[31] Choe YJ, Bae GR, Lee DH. No association between influenza A(H1N1)pdm09 vaccination and narcolepsy in South Korea: an ecological study. Vaccine. 2012;30(52):7439–7442. doi: https://doi.org/10.1016/j.vaccine.2012.10.030 23088885

[32] Duffy J, Weintraub E, Vellozzi C, DeStefano F, Vaccine Safety D. Narcolepsy and influenza A(H1N1) pandemic 2009 vaccination in the United States. Neurology. 2014;83(20):1823–1830. 25320099 10.1212/WNL.0000000000000987 PMC6563919

[33] Eurosurveillance editorial team. Swedish Medical Products Agency publishes report from a case inventory study on Pandemrix vaccination and development of narcolepsy with cataplexy. Euro Surveill. 2011;16(26):pii=19904. doi: https://doi.org/10.2807/ese.16.26.19904-en 21745441

[34] Barateau L, Dauvilliers Y. Recent advances in treatment for narcolepsy. Ther Adv Neurol Disord. 2019;12:1756286419875622. doi: https://doi.org/10.1177/1756286419875622 31632459 PMC6767718

[35] Kollb-Sielecka M, Demolis P, Emmerich J, Markey G, Salmonson T, Haas M. The European Medicines Agency review of pitolisant for treatment of narcolepsy: summary of the scientific assessment by the Committee for Medicinal Products for Human Use. Sleep Med. 2017;33:125–129. doi: https://doi.org/10.1016/j.sleep.2017.01.002 28449891

[36] EMA. Modafinil. https://www.ema.europa.eu/en/medicines/human/referrals/modafinil. 2011. Accessed October 3, 2023.

