# Supplementary material for: Epidemiology, patient characteristics, and treatment patterns of patients with narcolepsy in Sweden: a non-interventional study using secondary data
Source: Sleep Adv. 2024 Dec 24;5(1):zpae085. doi: 10.1093/sleepadvances/zpae085 (PMC11683588; doi:10.1093/sleepadvances/zpae085)
Supplement: zpae085_suppl_Supplementary_Materials [file zpae085_suppl_supplementary_materials.docx]

**Supplementary section**

**Title**: Epidemiology, patient characteristics, and treatment patterns of patients with narcolepsy in Sweden: A non-interventional study using secondary data

**Authors**: Anna Giertz^1^; Johan Mesterton^1,2^; Tanja Jakobsson^3^; Stephen Crawford^4^; Somraj Ghosh^4^; Anne-Marie Landtblom^5,6^

**Affiliations**: ^1^Quantify Research, Hantverkargatan 8, 112 21 Stockholm, Sweden; ^2^Department of Learning, Informatics, Management and Ethics, Medical Management Centre, Karolinska Institutet, Tomtebodavägen 18 A, 171 77, Stockholm, Sweden; ^3^Takeda Pharma AB, Lindhagensgatan 120, 112 51 Stockholm, Sweden; ^4^Takeda Development Center Americas, Inc., Cambridge, Massachusetts, United States; ^5^Department of Medical Sciences, Uppsala University, Uppsala, Sweden; ^6^Department of Biochemical and Clinical Sciences, Linköping University, Linköping, Sweden

**Corresponding author**: Anna Giertz (formerly Fornwall)

**Contact details**: Quantify Research, Hantverkargatan 8, 112 21 Stockholm, Sweden. [annafornwall@hotmail.com](mailto:annafornwall@hotmail.com).

**Supplementary Table 1. Comedications**

| **Drug Class** | **Drug name** | **ATC code** |
| --- | --- | --- |
| Wakefulness-promoting agents | Modafinil | N06BA07 |
| Stimulants | Amphetamine/ SR | N06BA01 |
|  | Dextroamphetamine | N06BA02 |
|  | Methylphenidate/SR | N06BA04 |
|  | Dexmethylphenidate | N06BA11 |
|  | Lisdexamfetamine | N06BA12 |
|  | Methamphetamine | N06BA03 |
|  | Atomoxetine | N06BA09 |
| Gamma-hydroxybutyrate | Sodium oxybate | N01AX11; N07XX04 |
| Histamine H3 receptors (H3Rs) antagonist | Pitolisant | N07XX11 |
| Hypnotics | Zolpidem/ CR | N05CF02 |
| Gamma-aminobutyric acid (GABA) receptor agonist | Baclofen | M03BX01 |
| Antidepressants | Venlafaxine | N06AX16 |
|  | Clomipramine | N06AA04 |
|  | Sertraline | N06AB06 |
|  | Citalopram | N06AB04 |
|  | Escitalopram | N06AB10 |
|  | Amitriptyline | N06AA09 |
| Melatonin receptor agonists | Melatonin | N05CH01 |
| Benzodiazepine derivatives | Flurazepam | N05CD01 |
|  | Estazolam | N05CD04 |
|  | Triazolam | N05CD05 |
|  | Lormetazepam | N05CD06 |
|  | Temazepam | N05CD07 |
|  | Midazolam | N05CD08 |
|  | Brotizolam | N05CD09 |
|  | Kvazepam | N05CD10 |
|  | Loprazolam | N05CD11 |
|  | Doxefazepam | N05CD12 |
|  | Cinolazepam | N05CD13 |
|  | Remimazolam | N05CD14 |
|  | Nimetazepam | N05CD15 |
| Other hypnotics and sedatives | Metakvalon | N05CM01 |
|  | Klometiazol | N05CM02 |
|  | Bromisoval | N05CM03 |
|  | Karbromal | N05CM04 |
|  | Skopolamin | N05CM05 |
|  | Porpiomazin | N05CM06 |
|  | Triklofos | N05CM07 |
|  | Klorvinylpentiol | N05CM08 |
|  | Valerianae radix | N05CM09 |
|  | Hexapropymat | N05CM10 |
|  | Bromider | N05CM11 |
|  | Apronal | N05CM12 |
|  | Valnoktamid | N05CM13 |
|  | Metylpentynol | N05CM15 |
|  | Niaprazin | N05CM16 |
|  | Dexemedetomidin | N05CM18 |
|  | Suvorexant | N05CM19 |

A wide range of medications from the pharmacologic types used in narcolepsy were included, subsequently medications not used in narcolepsy are also reported here.

**Supplementary Table 2. Prevalence of narcolepsy in specialist care in Sweden between 2015–2020**

| **Prevalence (per 100,000)** | **All patients** | | | **Males** | | | **Females** | | |
| --- | --- | --- | --- | --- | --- | --- | --- | --- | --- |
|  | Number of patients | Population at risk | Prevalence per 100,000 individuals | Number of patients | Population at risk | Prevalence per 100,000 individuals | Number of patients | Population at risk | Prevalence per 100,000 individuals |
| 2015 | 981 | 9,838,418 | 10.0 | 417 | 4,923,562 | 8.5 | 564 | 4,914,856 | 11.5 |
| 2016 | 1,309 | 9,967,637 | 13.1 | 543 | 4,997,278 | 10.9 | 766 | 4,970,359 | 15.4 |
| 2017 | 1,490 | 10,104,036 | 14.7 | 619 | 5,073,693 | 12.2 | 871 | 5,030,343 | 17.3 |
| 2018 | 1,641 | 10,215,309 | 16.1 | 680 | 5,133,164 | 13.2 | 961 | 5,082,145 | 18.9 |
| 2019 | 1,783 | 10,319,473 | 17.3 | 739 | 5,191,317 | 14.2 | 1,044 | 5,128,156 | 20.4 |
| 2020 | 1,762 | 10,378,483 | 17.0 | 732 | 5,222,532 | 14.0 | 1,030 | 5,155,951 | 20.0 |

**Supplementary Table 3. Incidence of narcolepsy in specialist care in Sweden between 2015–2020**

| **Incidence (per 100,000)** | **All patients** | | | **Males** | | | **Females** | | |
| --- | --- | --- | --- | --- | --- | --- | --- | --- | --- |
|  | Number of patients | Population at risk | Incidence per 100,000 individuals | Number of patients | Population at risk | Incidence per 100,000 individuals | Number of patients | Population at risk | Incidence per 100,000 individuals |
| 2015 | 96 | 9,737,559 | 1.0 | 41 | 4,866,363 | 0.8 | 55 | 4,871,196 | 1.1 |
| 2016 | 104 | 9,838,418 | 1.1 | 35 | 4,923,562 | 0.7 | 69 | 4,914,856 | 1.4 |
| 2017 | 93 | 9,967,637 | 0.9 | 38 | 4,997,278 | 0.8 | 55 | 4,970,359 | 1.1 |
| 2018 | 88 | 10,104,036 | 0.9 | 39 | 5,073,693 | 0.8 | 49 | 5,030,343 | 1.0 |
| 2019 | 85 | 10,215,309 | 0.8 | 37 | 5,133,164 | 0.7 | 48 | 5,082,145 | 0.9 |
